# Concomitant minimally invasive surgery for tricuspid valve papillary fibroelastoma and right lung cancer in an elderly patient: a case report and review of the literature

**DOI:** 10.1186/s13019-023-02431-x

**Published:** 2023-11-10

**Authors:** Yalin Wang, Neckwaree Aboo Bakar Shah Ameer Saheb, Firyuza Husanova, Weidong Li, Hongfei Xu

**Affiliations:** 1 Department of Nursing, School of Medicine, The First Affiliated Hospital of Zhejiang University, Hangzhou, China; 2 Department of Cardiovascular Surgery, School of Medicine, The First Affiliated Hospital of Zhejiang University, Hangzhou, China

**Keywords:** Papillary fibroelastoma, Video-assisted thoracic Surgery, Minimally invasive cardiac Surgery

## Abstract

**Background:**

It is very common for patients with newly diagnosed lung masses to have heart disease. However, papillary fibroelastomas (PFEs) of the tricuspid valve (TV) combined with lung cancer are rarely reported. It is thus unclear whether a two-stage surgery or concomitant surgery is optimal.

**Case presentation:**

We report the case of a 73-year-old Chinese male who was diagnosed with PFEs on the TV by transthoracic echocardiography (TTE) examination while being evaluated to undergo video-assisted thoracic surgery (VATS) for a right lower lung nodule. We resected both the PFEs and the lung nodule via right minithoracotomy. The surgery was uneventful, and histopathology reports confirmed PFEs of the TV and moderately to poorly differentiated squamous cell carcinoma. The patient recovered uneventfully, and there was no sign of tumor recurrence during 15 months of follow-up.

**Conclusions:**

We suggest that after careful evaluation, concomitant minimally invasive radical resection of primary lung cancer after cardiac PFE removal is an acceptable and safe treatment strategy and should be performed as soon as possible.

## Background

Cardiac tumors can be incidentally discovered during preoperative evaluation for lung cancer surgery. Treatment strategies include concomitant or two-stage surgery. PFEs account for 4.4–8% of primary cardiac tumors [1]. Approximately 15% of PFEs are located on the TV [2]. Although tricuspid PFEs are rare and predominantly asymptomatic, they may be associated with serious complications, namely, thromboembolic events [3]. Tricuspid PFEs combined with lung cancer are rarely reported, and conventional simultaneous operations usually need to be performed under median sternotomy. Here, we describe concomitant PFE resection under cardiopulmonary bypass and VATS lobectomy with selective lymphadenectomy through a single right thoracic incision to treat both diseases and thereby avoiding the adverse effects of median sternotomy, ischemia/reperfusion injury, repeated anesthesia, and pain without delaying lung cancer treatment or increasing the risk of pulmonary embolism.

## Case presentation

**Fig. 1:**
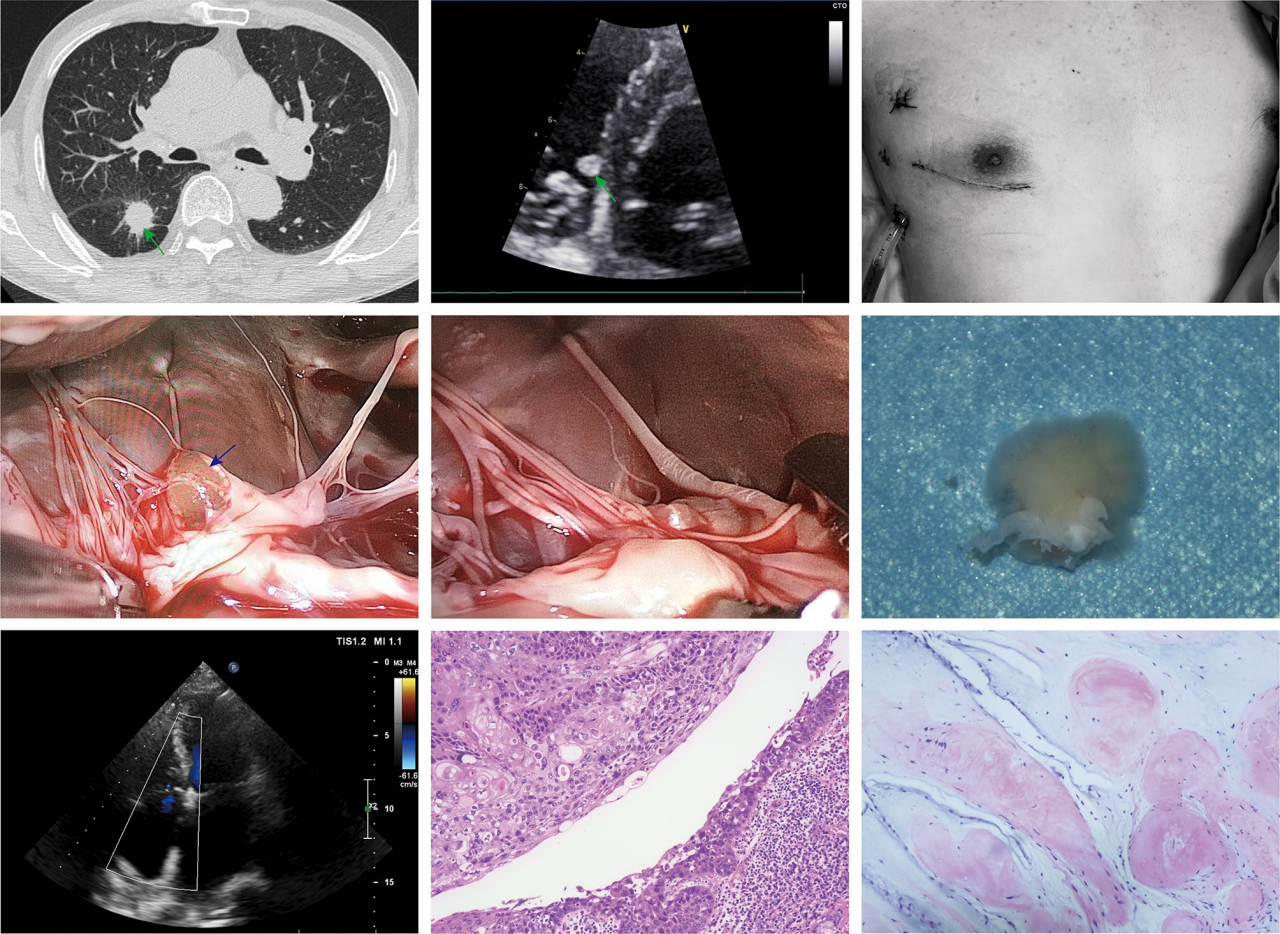
Concomitant surgery for tricuspid valve (TV) papillary fibroelastoma and right lung cancer.**a** Chest computed tomography revealed a right lower pulmonary nodule measuring 2.5 × 2 cm with burrs visible on the margin. **b** Transthoracic ultrasound revealed a 0.84 × 0.6 cm mass on the septal leaflet of the TV adjacent to the interventricular septum. **c** Operative incisions of the patient. **d** A spherical mass attached to the edge of the TV surrounded by chordae. **e** The mass was removed from the TV with the valve preserved. **f** Papillary fibroelastomas with an anemone-like appearance and villi on their surface when submerged in water. **g** Postoperative color Doppler echocardiography showed satisfactory result of tricuspid annuloplasty. **h** The pathological findings revealed squamous cell carcinoma (HE × 100). **i** Histological sections showing a cavity filled with mature collagen covered by a single layer of endocardial cells

A 73-year-old patient reported numbness in his fingers and was found to have a right lower lung nodule (2.5 × 2 cm) through annual computed tomography examination (Fig. 1a). The patient was being evaluated to undergo VATS. While undergoing TTE examination, a slightly hyperechoic mass was found on the TV and seemed attached to the interventricular septum by a short stalk (Fig. 1b), and tricuspid annular enlargement with moderate regurgitation was also observed. The mass was approximately 0.6 cm below the septal leaflet of the TV and appeared to swing slightly irrespective of leaflet movement. The size of the mass was 0.84 × 0.6 cm. Additional imaging tests, including transoesophageal echocardiography (TEE), that were performed immediately before the surgery confirmed the above findings.

The patient underwent complete anesthesia and was intubated with a double lumen endotracheal tube. The patient was positioned in the same position used for traditional right minithoracotomy, which is the right side elevated to an angle of 30 degrees. Following routine disinfection, draping and deflation of the right lung, the first 8-cm incision (Fig. 1c) was made along the 4th intercostal space lateral to the midclavicular line to access the pericardium. The second step was the release of heparin and cannulation of the femoral vein and artery, respectively. After opening the pericardium longitudinally on the right side anterior to the phrenic nerve and placing stay sutures, we placed a camera port in the third intercostal space at the anterior axillary line. The right atrium was then opened while the heart was beating under cardiopulmonary bypass, and a shunt that was used to drain blood from the superior vena cava through the femoral vein cannula allowed good exposure of the anatomy of the TV and right ventricle, as described in our previously published article [4]. The tumor was wrapped around the tricuspid septal leaf (Fig. 1d), and resected completely from its peduncle base, thus allowing proper valve preservation (Fig. 1e). The PFEs had an anemone-like appearance with villi on their surface when submerged in water (Fig. 1f). After tumor resection, a #28 flexible ring was placed on the valve’s annulus and sutured with 4 − 0 Prolene continuous sutures to our band using the annuloplasty technique [5]. After the repair of the TV was confirmed by TEE (Fig. 1g), the left lung ventilation was restored, protamine was released, and all cannulas were removed sequentially.

When the cardiac operating area was cleared, we used the proper VATS procedure to identify and resect the lung lesion with an endoscopic surgical stapler. As the lesion had a malignant appearance, we performed a complete right lower lobectomy with removed all the surrounding mediastinal lymph nodes (lymph nodes 2, 4, 7, and 9). All specimens were sent to the pathological lab, and the duration of the surgery was 3 h 30 min. The histopathology examination revealed squamous cell carcinoma (SCC) of the right lower lung (Fig. 1h) without tumor infiltration in the lymph nodes (including N1 station) and confirmed cardiac PFEs (Fig. 1i). The patient recovered uneventfully and was discharged 8 days after the operation. After 15 months of follow-up, no tumor recurrence was observed.

## Discussion

The number of elderly patients with simultaneous heart disease and lung tumors is increasing. In view of this situation, there is some controversy on the issue of staged surgery or simultaneous surgery [6]. Cheng et al. analyzed 536 patients who underwent simultaneous cardiac surgery and lung tumor resection and showed that the combined procedure had a low mortality rate and an acceptable complication rate [7]. Concerns have been raised about lymph node dissection in concomitant cardiac surgery and lung cancer resection under median sternotomy [8], and we presented simultaneous minimally invasive lateral thoracotomy as a surgery option in a previous case [9].

The first ever case of PFEs was incidentally found in ventricular septal defect repair in 1979 [10]. Their clinical symptoms are often nonspecific and usually covert, and, as a result, their diagnosis and surgical management are often delayed. Surgical intervention should be recommended if PFEs are symptomatic, mobile, or located in the left cardiac system [11]. In our case, the patient showed no specific symptoms, and the tumor was on the right side, with a size of 0.84 cm x 0.6 cm and relatively good mobility. It is suggested that right-sided lesions be observed and surgically excised only if they become symptomatic [12]. However, Guglielmo and colleagues advised that mobile lesions, regardless of size, should be surgically excised given the higher risk of thromboembolic complications [2], which was our key consideration for surgical resection.

Since we were already evaluating the patient before VATS, we decided to access the TV through the same incision to avoid median sternotomy surgery given patient’s old age. The use of cardioplegic arrest with aortic cross-clamping is the standard procedure for myocardial protection in cardiac tumor resection surgery. Due to the patient’s advanced age, we preferred to excise the PFEs using the on-pump beating-heart technique, as we described previously [13].

The histopathology examination revealed SCC of the right lower lung without tumor infiltration in the lymph nodes and confirmed the diagnosis of cardiac PFEs. The patient was followed for 15 months with no sign of SCC tumor or PFE recurrence. Finally, it is important to note that PFEs have a 1.6% recurrence rate [1], and this fact should not be ignored before considering surgery and during follow-up sessions.

## Conclusion

Cardiac tumors and lung cancer occasionally coexist. We suggest that after careful evaluation, radical resection of primary lung cancer after cardiac PFE surgery is an acceptable and safe option and should be implemented as soon as possible. We provide a therapeutic approach in which a a single intervention involving a right minithoracotomy was performed for such combined lesions.

## Data Availability

Not applicable.

## Abbreviations

PFEs: Papillary fibroelastomas
TTE: Transthoracic echocardiography
VATS: Video-assisted thoracic surgery
TV: Tricuspid valve

## References

[1] Tamin SS, Maleszewski JJ, Scott CG, Khan SK, Edwards WD, Bruce CJ, Oh JK, Pellikka PA, Klarich KW (2015). Prognostic and bioepidemiologic implications of Papillary Fibroelastomas. J Am Coll Cardiol.

[2] Actis Dato GM, Calia C, Lodo V, Fadde M, Cappuccio G, Italiano E, Addonizio M, Stefan AB, Centofanti P. A rare case of papillary fibroelastoma involving the tricuspid valve. A single center experience over a period of 22 years (1999–2021). Acta Chir Belg 2022 May 16:1–3. 10.1080/00015458.2022.2064625 35395925

[3] Mazur P, Kurmann R, Klarich KW, Dearani JA, Arghami A, Daly RC, Greason K, Schaff HV, Ahmad A, El-Am E, Sorour A, Bois MC, Viehman J, King KS, Maleszewski JJ, Crestanello JA. Operative management of cardiac papillary fibroelastomas. J Thorac Cardiovasc Surg. 2022 Jul 16:S0022-5223(22)00744-9. 10.1016/j.jtcvs.2022.06.022 35989118

[4] Wu S, Ma L, Li C, Ni Y (2019). Optimizing venous drainage for Minimal Access Right Atrial procedures. Ann Thorac Surg.

[5] Xu H, Davies H, Zheng J, Peng T, Ni Y (2019). Modified band annuloplasty technique for functional tricuspid regurgitation repair in patients with grossly dilated annuli: the three-suture junctional continuous suture band annuloplasty technique. J Card Surg.

[6] Tricard J, Milad D, Chermat A, Simard S, Lacasse Y, Dagenais F, Conti M (2021). Staged management of cardiac Disease and concomitant early Lung cancer: a 20-year single-center experience. Eur J Cardiothorac Surg.

[7] Cheng S, Jiang Y, Li X, Lu X, Zhang X, Sun D (2021). Perioperative outcomes of combined heart Surgery and lung Tumor resection: a systematic review and meta-analysis. J Cardiothorac Surg.

[8] Tsukioka K, Kono T, Takahashi K, Tominaga Y, Sano K (2020). Concomitant resection of Left Ventricular Hemangioma and Lung Cancer with Ground-Glass opacity. Ann Thorac Surg.

[9] Xu H, Tao T, Ma L, Li W, Ni Y (2020). Concomitant Surgery for aortic valve and Lung cancer patients in an elder. J Cardiothorac Surg.

[10] Lichtenstein HL, Lee JC, Stewart S (1979). Papillary Tumor of the heart: incidental finding at Surgery. Hum Pathol.

[11] Tsugu T, Nagatomo Y, Endo J, Kawakami T, Murata M, Yamazaki M, Shimizu H, Fukuda K, Mitamura H, Lancellotti P (2019). Multiple papillary fibroelastomas attached to left ventricular side and aortic side of the aortic valve: a report of new case and literature review. Echocardiography.

[12] Yee HC, Nwosu JE, Lii AD, Velasco M, Millman A (1997). Echocardographic features of papillary fibroelastoma and their consequences and management. Am J Cardiol.

[13] Li W, Zheng J, Zhao H, Xu H, Ni Y (2016). Beating-heart surgical treatment of tricuspid valve papillary fibroelastoma: a case report. Med (Baltim).

